# Dynamics of Ischemia/Reperfusion Injury Markers During Normothermic Liver Machine Perfusion

**DOI:** 10.1097/TXD.0000000000001728

**Published:** 2024-11-14

**Authors:** Aránzazu Caballero-Marcos, Luis Rodríguez-Bachiller, Alberto Baroja-Mazo, Álvaro Morales, Paloma Fernández-Cáceres, María Fernández-Martínez, Fernando DíazFontenla, Enrique Velasco, Ainhoa Fernández-Yunquera, Benjamin Díaz-Zorita, Sergio Cortese, José María Pérez-Peña, Arturo Colón-Rodríguez, Mario Romero-Cristóbal, José Manuel Asencio, Rafael Bañares, José Ángel López-Baena, Magdalena Salcedo-Plaza

**Affiliations:** 1 Hepatology and Liver Transplantation Unit, Hospital General Universitario Gregorio Marañón, Universidad Complutense, Madrid, Spain.; 2 Centro de Investigación Biomédica en Red de Enfermedades Hepáticas y Digestivas (CIBERehd), Barcelona, Spain.; 3 Transplant and Hepatobiliopancreatic Surgery Unit, Department of General and Digestive Surgery, Hospital General Universitario Gregorio Marañón, Madrid, Spain.; 4 Biomedical Research Institute of Murcia (IMIB-Arrixaca-UMU), Murcia, Spain.; 5 Department of Anesthesiology, Reanimation and Intensive Care, Hospital General Universitario Gregorio Marañon, Madrid, Spain.

## Abstract

**Background.:**

A comprehensive mechanistic assessment of normothermic machine perfusion (NMP) is an essential step toward identifying biomarkers to assess liver viability. Although some studies have evaluated the effect of NMP on inflammation markers, there are other key pathological mechanisms involved in ischemia/reperfusion injury (IRI) that have not yet been evaluated.

**Methods.:**

Eight human donor livers preserved by NMP were included to analyze IRI during preservation. Concentrations of several biomarkers involved in different biological processes of IRI were measured in the perfusate.

**Results.:**

Perfusate levels of intercellular adhesion molecule 1, P-selectin, vascular cell adhesion molecule 1, metalloproteinase with thrombospondin motif type 1, member 13, phospholipase A2 group VII, and syndecan-1 progressively increased during NMP. Noteworthy, perfusate lactate levels showed a strong correlation with C-X-C motif chemokine ligand 10 (*P* = 0.001), intercellular adhesion molecule 1 (*P* = 0.01), and urokinase plasminogen activator (*P* = 0.001).

**Conclusions.:**

Perfusate lactate correlates with the main underlying biological mechanisms occurring in the NMP environment. Moreover, several IRI biomarkers accumulate during NMP, which may limit the extent of the benefits of this technology.

Liver perfusion machines have emerged as an innovative strategy for organ preservation and for reconditioning and viability assessment of available organs.^1^ However, despite its potential utility, there are critical aspects of ex vivo liver machine perfusion that have not been completely studied. Normothermic machine perfusion (NMP) tries to rebalance cellular metabolism by maintaining the organ at physiological temperature and also supplying oxygen and nutrients. Although these conditions may allow viability assessment before using these organs for liver transplantation (LT), a comprehensive mechanistic assessment of NMP is still lacking. Moreover, this is an essential step toward identifying biomarkers to assess and predict liver function and viability after LT.

Ischemia/reperfusion injury (IRI)^2^ is a well-described biphasic phenomenon that occurs after oxygenated reperfusion of ischemic tissues. IRI involves several mediators and pathological processes, being liver sinusoidal endothelial cells particularly susceptible.^3^ In fact, liver endothelium damage occurring during cold preservation is the initial factor leading to hepatic IRI,^3^ determining hepatic microcirculatory dysfunction, platelet activation, and upregulation of adhesion molecules that further promote liver immune cells infiltration, inducing additional injury.^3^

Although some studies have evaluated the effect of NMP on inflammation markers,^4-8^ other key pathological mechanisms involved in IRI, such as endothelial activation, have not yet been evaluated. A more comprehensive assessment is essential to optimize the potential prognostic value of IRI biomarkers, which could significantly improve organ assessment before LT. Hence, this study aimed to investigate the dynamics of markers of different processes involved in IRI during NMP of human livers and their association with currently used viability markers. Herein, we present preliminary findings from the initial cohort of human donor livers preserved by NMP in our center.

## PATIENTS AND METHODS

### Study Design

We conducted a single-center prospective observational study to analyze IRI of organs during NMP preservation. The secondary endpoint consisted of an analysis of the correlation of standard viability markers with IRI biomarkers. We prospectively included human donor livers preserved by NMP (OrganOx metra) between February 2022 and May 2023.

Transplanted patients gave written informed consent for using samples and clinical data for study purposes. The study was conducted in accordance with the ethical guidelines of the Declaration of Helsinki. Approval from the local ethics committee was obtained before starting the study.

### Donor Selection and Organ Management

Donor information was obtained from the data provided by the National Transplant Organization during the donation process. Eligibility for liver donation was assessed according to our institutional policy and national guidelines. Variables included age, sex, body mass index, cause of death, liver function tests at the time of procurement, comorbidities (diabetes mellitus, arterial hypertension, cardiovascular disease, renal disease, hemodialysis, and dyslipidemia), use of inotropic agents, history of cardiac arrest or hemodynamic instability, intensive care unit length of stay, and hepatitis B and hepatitis C virus serological status.

Livers were stored at 4 °C and sent to our center for preparation for bench surgery and then perfused in the machine (see below).

### Normothermic Machine Perfusion

The indication for NMP at the time of procurement was due to concerns about the quality or condition of the liver based on significant fatty infiltration, liver macroscopic appearance, advanced donor age, or previous medical conditions in most cases (n = 7; 87.5%). Only in 1 case, the decision was based on a predicted excessively prolonged cold ischemia time.

NMP was initiated using the OrganOx Metra liver perfusion device (OrganOx Limited, Oxford, United Kingdom) as described and published previously.^9,10^ Blood gas analysis was performed every 15 min for the first hour and every 60 min thereafter. Perfusate pH, glucose, aspartate aminotransferase, alanine aminotransferase (ALT), lactate, and bile production were collected hourly. Arterial and portal flow were also monitored at the same time points.

### Perfusate Analysis

To assess IRI in NMP organs, we measured the concentrations of several biomarkers in perfusate samples obtained during the NMP (hourly after NMP initiation until the end of the perfusion). Baseline samples were collected 1 h after starting NMP. To evaluate the physiopathological processes involved, we analyzed the following markers: inflammatory response and cell recruitment (C-C motif chemokine ligand [CCL] 2, CCL3, CCL4, C-X-C motif chemokine ligand [CXCL] 10, interleukin-18 and matrix metalloproteinase 9); endothelial activation and cell migration (E-selectin, intercellular adhesion molecule 1 [ICAM-1], P-selectin and vascular cell adhesion molecule 1); thrombogenesis and microcirculatory disturbances (metalloproteinase with thrombospondin motif type 1, member 13, von Willebrand factor and phospholipase A2 group VII); coagulation and fibrinolysis (coagulation factor III, tissue factor pathway inhibitor, thrombomodulin and serpin E1); liver injury and repair processes (urokinase plasminogen activator [uPA], vascular endothelial growth factor receptor 1); and degradation of the endothelial glycocalyx (syndecan-1). Biomarkers concentrations were assessed using the Luminex multiplex assay (R&D Systems, Minneapolis, MN) and measured with the MagPix System (Luminex, Austin, TX) at Murcia’s BioHealth Research Institute.

### Statistical Analysis

Quantitative variables are expressed as median (interquartile range [IQR]) and qualitative variables as n (%). Categorical data were analyzed using the chi-square test or the Fisher exact test. Quantitative variables were compared using the Mann-Whitney *U* test. Pearson’s correlation was used to evaluate the association between quantitative variables. A 2-tailed *P* value of <0.05 was considered statistically significant. Statistical analysis was performed using STATA Version 13.0. Due to the exploratory nature of this study, the sample size was not calculated.

## RESULTS

### Assessment of Liver Viability During NMP

During the study period, 8 livers were preserved by NMP. Median NMP time was 565.00 min (IQR, 465.00–697.50). During NMP, liver viability was evaluated by macroscopic appearance, arterial and portal flow, lactate clearance, pH, glucose levels, aspartate aminotransferase, ALT, and bile production. Five of 8 liver grafts preserved with NMP were considered suitable for transplantation (62.5%). Demographic and clinical characteristics of the donors and recipients, as well as LT outcomes, are shown in **Tables S1 and S2** (**SDC,** http://links.lww.com/TXD/A713). No significant differences were found between transplanted and discarded livers after NMP in terms of donor age (*P* = 1.00), body mass index (*P* = 0.25), type of donation (*P* = 0.46), and donor cause of death (*P* = 1.00). Similarly, NMP time was similar between transplanted and discarded livers (*P* = 0.65). Besides, discarded livers proceeded from donors who showed greater levels of ALT compared with transplanted livers, although without reaching statistical significance (199.00 U/L [IQR, 34.00–364.00] versus 81.00 U/L [IQR, 75.00–88.00], *P* = 0.70) and with a more frequent history of heart disease (100% versus 0%, *P* = 0.01).

Discarded livers showed a poorer lactate clearance during NMP, with higher lactate levels (6.70 mmol/L [IQR, 3.10–9.10] versus 1.30 mmol/L [IQR, 0.90–1.70], *P* = 0.05) and lower pH values (7.15 [IQR, 7.14–7.25] versus 7.26 [IQR, 7.26–7.39], *P* = 0.05) at the end of the perfusion. No additional differences were observed in the rest of the viability parameters determined in the perfusate (**Table S3, SDC,** http://links.lww.com/TXD/A713). Considering vascular flows, they were constant and within physiologic range during the preservation period in both groups (**Table S3, SDC,** http://links.lww.com/TXD/A713).

### Changes in IRI Biomarkers During NMP

**FIGURE 1.**
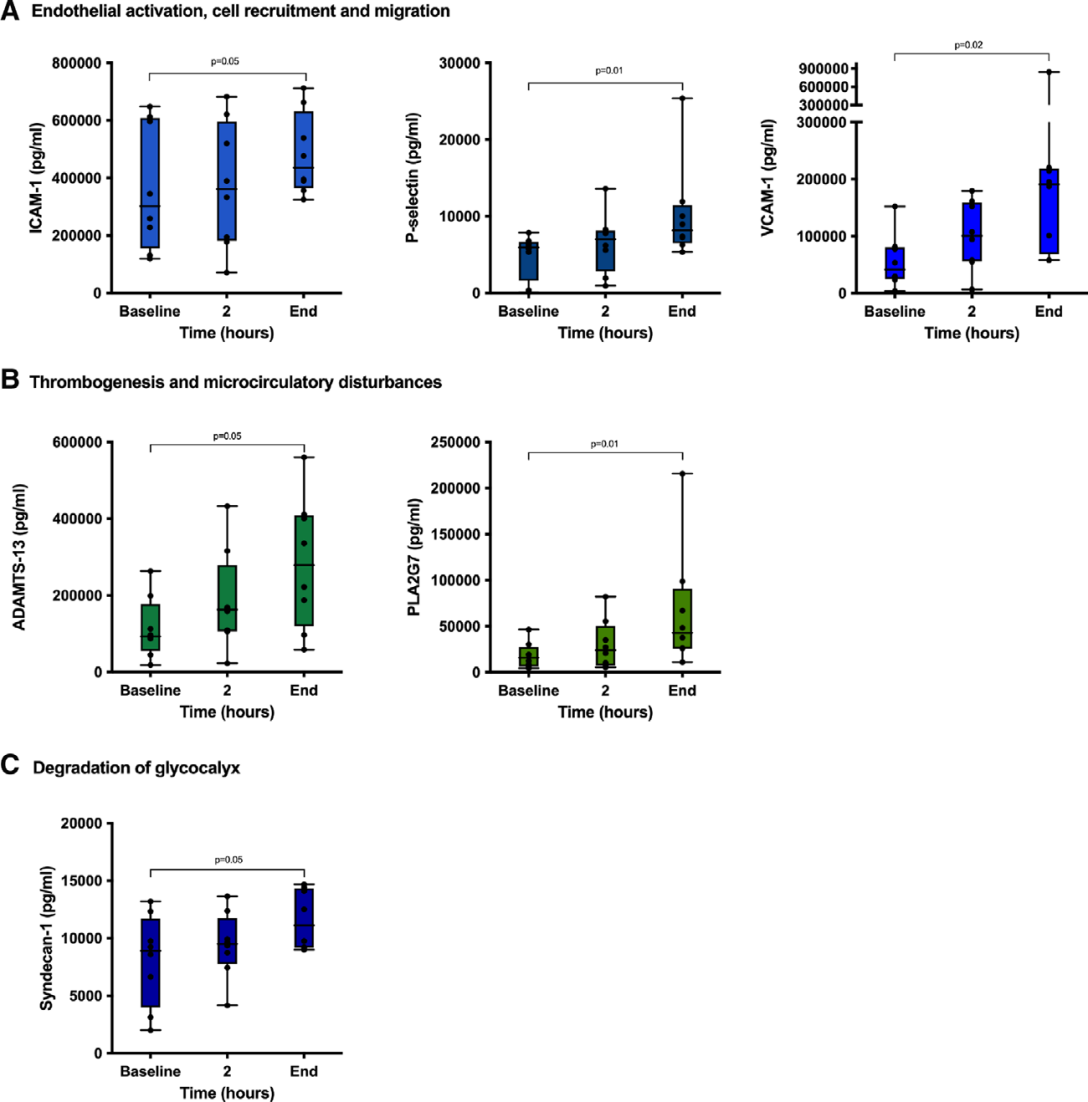
Changes from baseline of IRI biomarkers during NMP. A, Markers of endothelial activation, cell recruitment and migration. B, Markers of thrombogenesis and microcirculatory alterations. C, Endothelial glycocalyx degradation. Bars represent the median and the error bars represents the IQR. Baseline samples were collected 1 h after starting perfusion. ADAMTS13, disintegrin and metalloproteinase with thrombospondin motif type 1, member 13; ICAM-1, intercellular adhesion molecule 1; IQR, interquartile range; IRI, ischemia/reperfusion injury; NMP, normothermic machine perfusion; PLA2G7, phospholipase A2 Group VII; VCAM-1, vascular cell adhesion molecule-1.

Figure 1 shows levels of IRI biomarkers at different time points during preservation and the percentage of change from the baseline. Significant differences were observed between baseline and end-of-preservation levels of biomarkers of endothelial activation, cell recruitment and migration, thrombogenesis and microcirculatory alterations, and endothelial glycocalyx degradation (Figure 1). Moreover, NMP time showed a strong correlation with several of these markers (P-selectin: *r* = 0.89, *P* = 0.003; vascular cell adhesion molecule 1: *r* = 0.80, *P* = 0.02; ADAMTS13: *r* = 0.77, *P* = 0.02).

**FIGURE 2.**
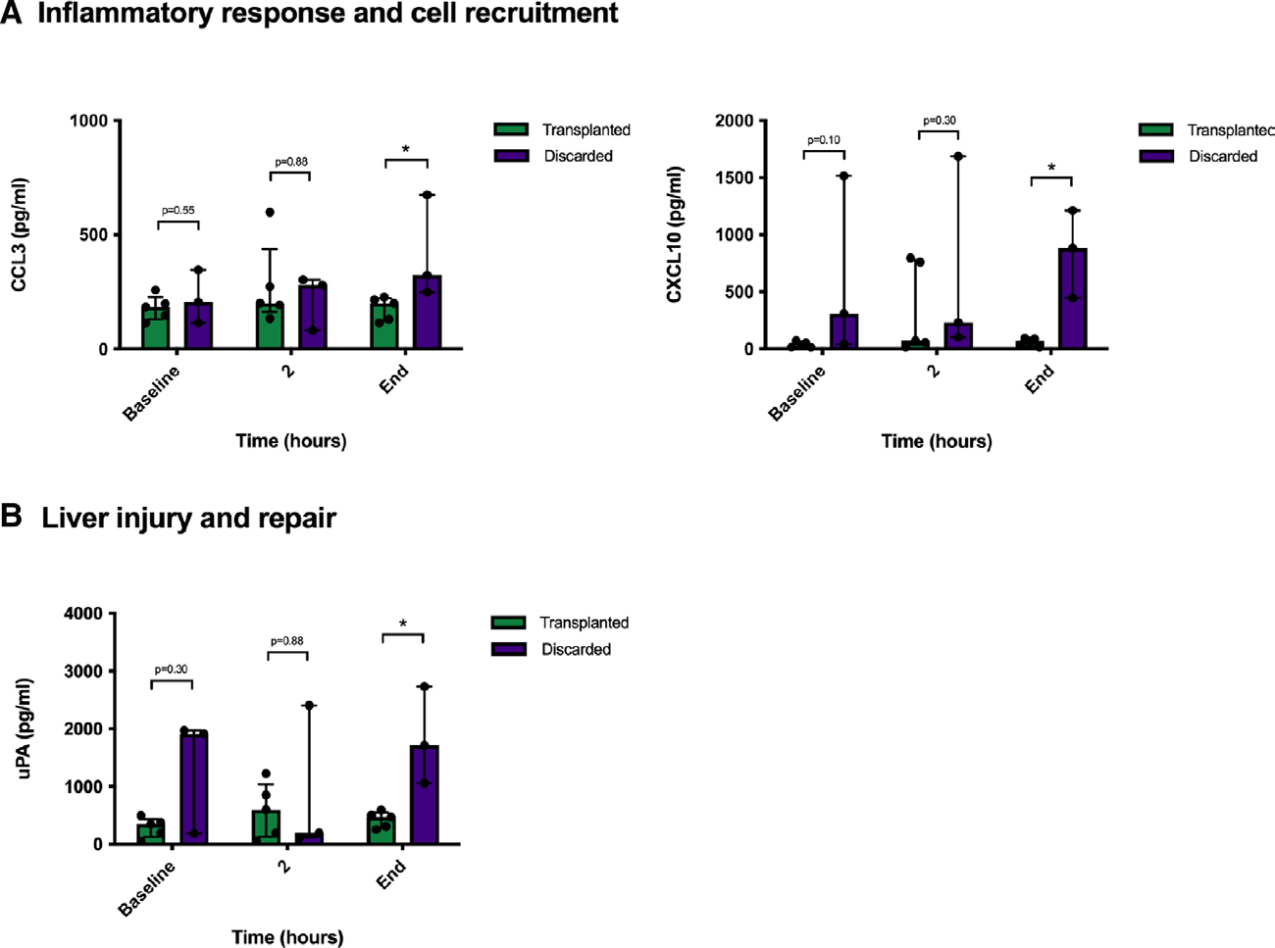
Changes in IRI markers in perfusate according to the result of the viability assessment. A, Markers of inflammatory response and cell recruitment. B, Liver injury and repair markers. Bars represent the median and the error bars represents the IQR. CCL, C-C motif chemokine ligand; CXCL, C-X-C motif chemokine ligand; IQR, interquartile range; IRI, ischemia/reperfusion injury; uPA: urokinase plasminogen activator.

Furthermore, we assessed differences in IRI biomarker levels according to organ viability. As shown in Figure 2, higher levels of inflammatory response and cell recruitment markers, such as CCL3 and CXCL10, were observed at the end of perfusion in discarded organs. Moreover, higher levels of liver injury and repair markers, such as uPA, were also observed in discarded livers (Figure 2). Additionally, greater nonsignificant levels of other IRI biomarkers were observed in the perfusate of organs considered unsuitable for transplantation (Figure 2).

### Correlation Between Standard Viability Markers and IRI Biomarkers

**FIGURE 3.**
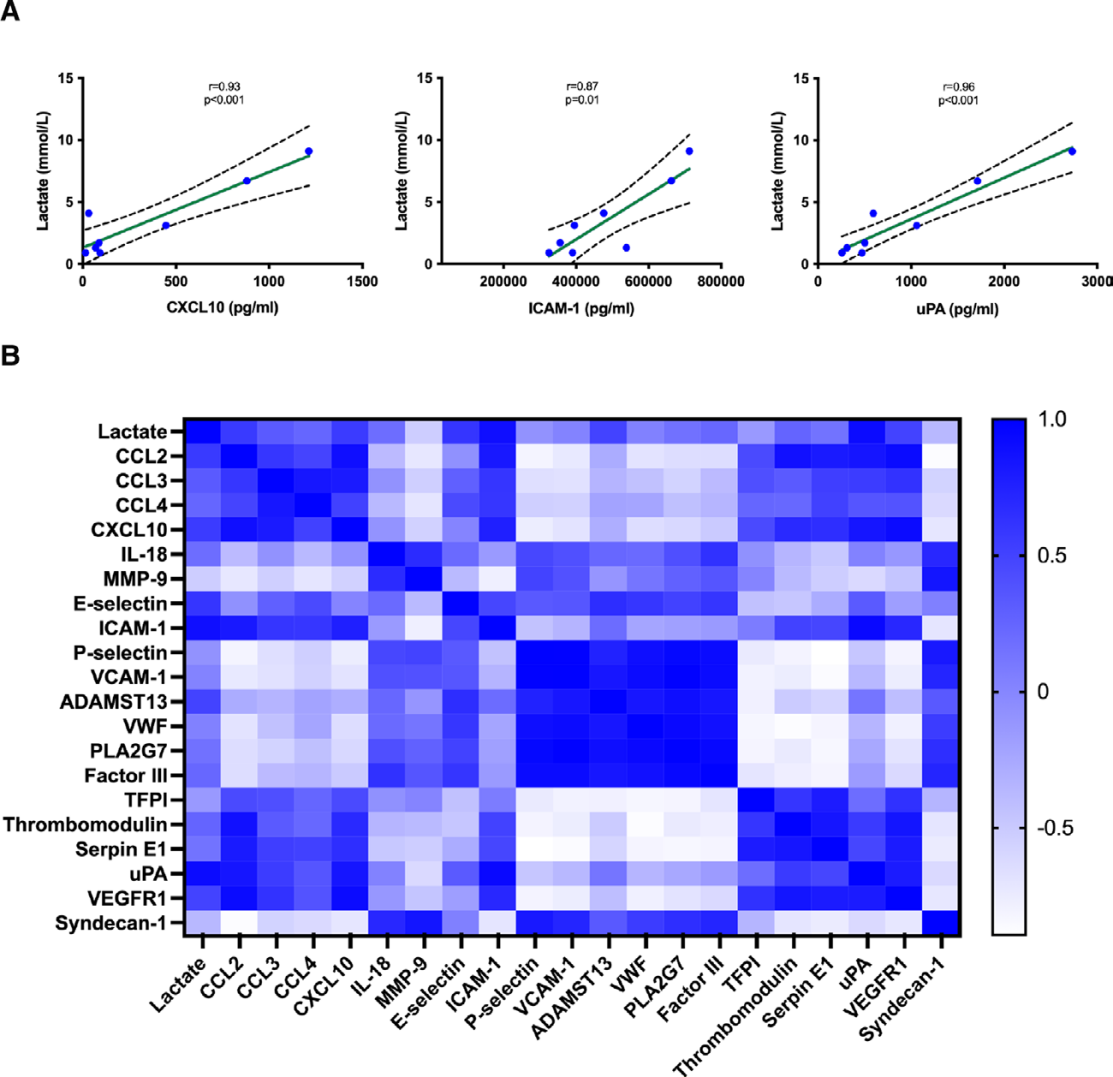
A, Correlation between perfusate lactate levels and IRI biomarkers at the end of NMP. The 2 confidence bands define the 95% confidence interval. B, Correlation matrix of perfusate lactate levels with IRI biomarkers at the end of NMP. The color scale represents the degree of correlation from –1 (white) to 1 (blue). ADAMTS13, disintegrin and metalloproteinase with thrombospondin motif type 1, member 13; CCL, C-C motif chemokine ligand; CXCL, C-X-C motif chemokine ligand; ICAM-1, intercellular adhesion molecule 1; IL, interleukin; IRI, ischemia/reperfusion injury; MMP-9, matrix metalloproteinase-9; NMP, normothermic machine perfusion; TFPI, tissue factor pathway inhibitor; uPA, urokinase plasminogen activator; VCAM-1, vascular cell adhesion molecule 1; VEGFR1, vascular endothelial growth factor receptor 1; VWF, von Willebrand factor.

To assess the potential association between current laboratory parameters used for viability assessment during NMP and the underlying pathophysiological mechanisms, we investigated its correlation with IRI biomarkers. Noteworthy, perfusate lactate levels showed a strong correlation with markers of inflammatory response and cell recruitment (CXCL10: *r* = 0.93, *P* = 0.001), endothelial activation (ICAM-1: *r* = 0.87, *P* = 0.01), and liver injury and repair (uPA: *r* = 0.96, *P* = 0.001) at the end of NMP (Figure 3A). Although other potentially relevant correlations were observed, they did not reach statistical significance (Figure 3B).

## DISCUSSION

In this study, we comprehensively analyzed the biological processes and mechanisms involved in IRI during NMP, a relevant fact that has not been fully elucidated. Moreover, we tried to assess their correlation with the viability assessment parameters currently used.

IRI is a common cause of morbidity and mortality after LT^11,12^ that involves many cell types and pathways.^13^ When blood flow is obstructed or diminished, reduced oxygen availability affects mitochondrial function, initiating cells anaerobic respiration, which increases lactic acid release to the extracellular space.^14^ Lactate accumulation has been associated in experimental models with an increased expression of adhesion molecules, and with reduction in nitric oxide synthesis resulting in enhancement of neutrophil adhesion, hepatic stellate cell contraction, and platelet aggregation.^15-17^ Not surprisingly, lactate clearance is commonly used as a surrogate marker for IRI and is one of the most widely accepted indicators of liver function during NMP.^18^ Poorer lactate clearance during NMP was one of the main and most decisive criteria used for viability assessment in our study. However, little is known about the potential association between lactate perfusate levels and the pathological processes resulting from IRI that occur during NMP. Interestingly, our data show that lactate levels significantly correlate with several IRI biomarkers involved in the inflammatory response and cellular recruitment (CXCL10), endothelial activation (ICAM-1), and processes of liver injury and repair (uPA). Therefore, our results confirm that, despite its limitations as a viability marker,^19,20^ perfusate lactate correlates with the main underlying biological mechanisms occurring in the NMP environment. Furthermore, discarded livers showed a greater degree of IRI, with higher levels of inflammatory response, cell recruitment, and liver injury and repair markers compared with transplanted livers. This observation also supports the association between lactate concentrations and the main IRI pathological events. On the contrary, other standard parameters commonly used for viability assessment, such as glucose metabolism or transaminases, do not appear to appropriately reflect the biological processes occurring during NMP.

Another relevant finding of our investigation is the increase observed in several IRI biomarkers during NMP. Although the accumulation of inflammatory markers has already been described,^5,7,8^ our study identifies a significant accumulation of markers involved in other pathophysiological mechanisms, which may play a relevant role. Interestingly, we observed the accumulation of endothelial activation, thrombogenesis, microcirculatory disturbances, and glycocalyx degradation markers. This is of particular importance because the accumulation of these mediators during the preservation period may limit the extent of benefit of this technology. In vivo, cytokines, damage-associated molecular patterns, and other mediators experience rapid degradation in circulation due to the influence of plasma enzymes and renal excretion.^21,22^ However, NMP devices do not have these clearance mechanisms, potentially leading to the extended presence and activity of inflammatory molecules throughout the preservation period. This is of particular relevance given that previous studies have demonstrated that damage-associated molecular patterns released during liver machine perfusion^23^ and static cold storage^24^ have a deleterious effect on the organ by triggering a self-perpetuating cycle of inflammation and additional cell damage. Therefore, our results support the need for interventions to attenuate the detrimental effect of the accumulation of these mediators to potentially improve outcomes with NMP technology. In this regard, our group has shown that defibrotide inhibits the activation of endothelial cells observed after static cold storage,^25^ emerging as a promising therapeutic agent to mitigate the potential negative effect of the accumulation of the above-mentioned mediators. Additionally, other strategies such as the integration of hemodialysis filters into normothermic perfusion systems are also being explored.^26,27^

Furthermore, our findings could contribute to the identification of perfusate biomarkers for assessing organ viability before LT. Although correlation with clinical outcomes is crucial, organs deemed unsuitable for LT exhibited elevated levels of several IRI markers. Of particular interest among these markers is CXCL10, a chemokine that targets activated T cells and natural killer cells expressing CXCR3.^28^ This chemokine plays a critical role in signaling in liver inflammation and the pathophysiology of IRI.^29^ Thus, it regulates response against liver IR by promoting proinflammatory gene induction, and the balance between CXCL10 and interleukin-10 determines the nature of response to IR insult and the development of hepatocellular injury.^29^ Moreover, the functional role of CXCL10 occurs early after reperfusion (within 6 h), rather than during the chronic phase of IRI.^29^ This makes it a suitable marker for organ viability assessment during NMP. However, since the evaluation of organ viability inherently includes a subjective component, these results should be interpreted with caution and correlated with recipient outcomes in future studies.

Limitations of our study include the low number of cases, which may have limited the ability to detect additional relevant differences in certain parameters and precluded differentiation between donation after circulatory death and donation after brain death donors. Moreover, a larger sample size is needed to validate the observed trends further and provide more reliable insights into clinical implications. Furthermore, because we did not determine levels of the IRI markers in the perfusate before the start of NMP, we cannot exclude the possibility that their presence might have influenced baseline values, although a significant impact seems unlikely. Finally, we could not assess histological changes to investigate the association between changes in IRI biomarkers and histological manifestations. However, histological changes observed after NMP have been widely described^4,7,30^; moreover, our study was focused on the analysis of soluble markers that could facilitate the identification of reliable viability markers in the future.

In conclusion, our results show an association between the biological processes involved in liver IRI during NMP and one of the most relevant currently used viability markers. These findings offer potential clinical utility while awaiting the identification of more reliable viability markers. Moreover, our results emphasize the need for interventions to reduce the potential detrimental effect of the accumulation of several mediators during NMP. Further research is needed to explore these findings in a larger sample size and to assess their correlation with clinical outcomes.

## ACKNOWLEDGMENTS

The authors thank Ana Hernández and Laura Secadas for their contribution to sample collection, sample processing, and transportation.

## Supplementary Material


