# Supplementary material for: Dynamics of Ischemia/Reperfusion Injury Markers During Normothermic Liver Machine Perfusion
Source: Transplant Direct. 2024 Nov 14;10(12):e1728. doi: 10.1097/TXD.0000000000001728 (PMC11567704; doi:10.1097/TXD.0000000000001728)
Supplement: Supplementary file 1 [file txd-10-e1728-s001.pdf]

	Transplanted		Discarded		
Donor characteristics	n=5		n=3		p
Age (years)	54.00	(44.00-58.00)	53.00	(43.00-65.00)	1.00
BMI (kg/m^2)	25.22	(24.49-27.76)	29.99	(26.03-33.95)	0.25
Donor type (category 3 DCD)	4	(80.00)	1	(33.33)	0.46
Cause of death (CVA)	2	(40.00)	1	(33.33)	1.00
ICU lenght of stay (hours)	72.00	(48.00-192.00)	72.00	(5.00-164.00)	0.55
Cardiac arrest	2	(40.00)	2	(66.67)	0.43
Time of cardiac arrest (minutes)	47.50	(45.00-50.00)	16.50	(8.00-25.00)	0.12
Arterial hypertension	2	(40.00)	1	(33.33)	1.00
Diabetes mellitus	0	(0.00)	1	(33.33)	0.38
Dyslipidaemia	0	(0.00)	2	(66.67)	0.11
Heart disease	0	(0.00)	3	(100.0)	0.01
ALT (U/L)	81.00	(75.00-88.00)	199.00	(34.00-364.00)	0.70
GGT (U/L)	56.00	(54.00-69.00)	63.00	(31.00-240.00)	0.88
AP (U/L)	69.00	(68.00-118.00)	90.00	(71.00-109.00)	0.70
Total bilirubin (mg/dL)	0.50	(0.48-0.90)	0.84	(0.28-1.40)	1.00
Na (mEq/L)	146.00	(142.00-150.00)	154.50	(145.00-164.00)	0.44

Table S1. Donor characteristics according to the result of the viability assessment and the preservation type (NMP and SCS). Data are expressed as median (IQR) or n (%). NMP: normothermic machine perfusion; SCS: static cold storage; BMI: body mass index; DCD: donation after circulatory death; CVA: cerebrovascular accident; ICU: intensive care unit; ALT: alanine aminotransferase; AST: aspartate aminotransferase; GGT: gamma-glutamyl transpeptidase; AP: alkaline phosphatase.

	NMP	
**Recipient characteristics**	**n=5**	
Baseline characteristics		
Age (years)	61.00	(59.00-67.00)
Sex (male)	4	(80.00)
MELD	17	(12-22)
Child-Pugh	8	(7-10)
Transplantation and post-transplant outcomes		
Reperfusion syndrome	0	(0.00)
Inotropic support	3	(60.00)
ICG PDR	16.60	(15.05-20.00)
ICG PDR ≥ 10	5	(100.00)
ALT peak (U/L)	729.00	(468.00-1407.00)
24 hours ALT (U/L)	729.00	(399.00-868.00)
24 hours INR	1.65	(1.48-1.87)
Early complication	1	(20.00)
Primary nonfunction	0	(0.00)
AKI	3	(60.00)
Acute rejection	2	(40.00)

Table S2. Recipient characteristics and post-transplant outcomes. Postreperfusion syndrome and primary non-function were evaluated according to stablished definitions. Early postoperative complications included primary non-function, biliary or vascular complications (such as biliary leak or hepatic artery thrombosis) and abdominal bleeding occurring during the first month after LT. Data are expressed as median (IQR) or n (%). NMP: normothermic machine perfusion; ALT: alanine aminotransferase; ICG PDR: plasma disappearance rate of indocyanine green; AKI: acute kidney injury.

	NMP				
	Transplanted		Discarded		
**Parameter**	**n=5**		**n=3**		**p**
**NMP time (minutes)**	570.00	(560.00-690.00)	540.00	(360.00-1140.00)	0.65
**2 hours portal flow (L/min)**	1.00	(1.00-1.20)	1.10	(1.00-1.20)	0.82
**4 hours portal flow (L/min)**	1.00	(1.00-1.00)	1.10	(1.00-1.20)	0.46
**2 hours arterial flow (L/min)**	0.40	(0.40-0.40)	0.30	(0.20-0.40)	0.15
**4 hours arterial flow (L/min)**	0.40	(0.40-0.50)	0.45	(0.30-0.60)	0.84
**Bile production (ml/h)**	10.00	(8.50-10.00)	5.00	(0.00-10.00)	0.42
**Baseline lactate (mmol/L)**	2.00	(1.10-4.80)	2.50	(2.30-5.70)	0.30
**Baseline pH**	7.28	(7.11-7.34)	7.07	(7.04-7.14)	0.10
**Baseline glucose (mg/dL)**	88.50	(10.00-240.00)	116.00	(19.00-134.00)	1.00
**Baseline ALT (U/L)**	1603.00	(930.00-2425.00)	909.00	(613.00-4356.00)	0.65
**Baseline AST (U/L)**	1614.00	(1118.00-3664.00)	743.00	(525.00-5573.00)	0.46
**2 hours lactate (mmol/L)**	1.30	(0.70-1.40)	2.90	(1.30-3.30)	0.13
**2 hours pH**	7.35	(7.27-7.36)	7.27	(7.26-7.28)	0.29
**2 hours glucose (mg/dL)**	39.00	(4.00-227.00)	145.00	(133.00-147.00)	0.65
**2 hours ALT (U/L)**	1627.00	(979.00-3894.00)	808.00	(720.00-4782.00)	0.46
**2 hours AST (U/L)**	1703.00	(1246.00-4667.00)	887.00	(528.00-6773.00)	0.30
**2 hours lactate < 2.2 mmol/L**	4	(80.00)	1	(33.33)	0.19
**2 hours lactate < 1.7 mmol/L**	4	(80.00)	1	(33.33)	0.19
**End of perfusion lactate (mmol/L)**	1.30	(0.90-1.70)	6.70	(3.10-9.10)	0.05
**End of perfusion pH**	7.26	(7.26-7.39)	7.15	(7.14-7.25)	0.05
**End of perfusion glucose (mg/dL)**	73.50	(6.00-147.00)	149.00	(115.00-204.00)	0.29
**End of perfusion ALT (U/L)**	1659.00	(1078.00-2873.00)	2684.00	(1244.00-4972.00)	0.65
**End of perfusion AST (U/L)**	1866.00	(1406.00-5175.00)	3389.00	(872.00-10190.00)	0.88

Table S3. Comparison of NMP preserved organs according to the result of the viability assessment. Data are expressed as median (IQR) or n (%). ALT: alanine aminotransferase; AST: aspartate aminotransferase; LDH: lactate dehydrogenase.
